# Rapid divergence of codon usage patterns within the rice genome

**DOI:** 10.1186/1471-2148-7-S1-S6

**Published:** 2007-02-08

**Authors:** Huai-Chun Wang, Donal A Hickey

**Affiliations:** 1 Department of Mathematics and Statistics, Dalhousie University, Halifax, Nova Scotia, B3H 2G1, Canada; 2 Department of Biology, Concordia University, 7141 Sherbrooke West, Montréal, Québec, H4B 1R6, Canada

## Abstract

**Background:**

Synonymous codon usage varies widely between genomes, and also between genes within genomes. Although there is now a large body of data on variations in codon usage, it is still not clear if the observed patterns reflect the effects of positive Darwinian selection acting at the level of translational efficiency or whether these patterns are due simply to the effects of mutational bias. In this study, we have included both intra-genomic and inter-genomic comparisons of codon usage. This allows us to distinguish more efficiently between the effects of nucleotide bias and translational selection.

**Results:**

We show that there is an extreme degree of heterogeneity in codon usage patterns within the rice genome, and that this heterogeneity is highly correlated with differences in nucleotide content (particularly GC content) between the genes. In contrast to the situation observed within the rice genome, *Arabidopsis *genes show relatively little variation in both codon usage and nucleotide content. By exploiting a combination of intra-genomic and inter-genomic comparisons, we provide evidence that the differences in codon usage among the rice genes reflect a relatively rapid evolutionary increase in the GC content of some rice genes. We also noted that the degree of codon bias was negatively correlated with gene length.

**Conclusion:**

Our results show that mutational bias can cause a dramatic evolutionary divergence in codon usage patterns within a period of approximately two hundred million years.

The heterogeneity of codon usage patterns within the rice genome can be explained by a balance between genome-wide mutational biases and negative selection against these biased mutations. The strength of the negative selection is proportional to the length of the coding sequences. Our results indicate that the large variations in synonymous codon usage are not related to selection acting on the translational efficiency of synonymous codons.

## Background

Synonymous codon usage patterns can vary significantly among genomes [1,2]. In addition, one can also observe differences in synonymous codon usage among different genes within a single genome (e.g., [3,4]). For prokaryotes and unicellular eukaryotes such as yeast, the variation in codon usage within a genome is thought to be due to natural selection acting to optimize protein production [5-7]. Specifically, the most highly expressed genes use codons that are complementary to the most abundant tRNA anticodons (e.g., [8,9]). For multicellular eukaryotes, such as *Drosophila melanogaster *and *Caenorhabditis elegans*, there is also some evidence that codon bias might be caused by selection for translational efficiency [10,11]. For the majority of multicellular organisms, however, it has been difficult to explain codon usage variation within a genome in terms of natural selection. Instead, the codon usage in mammalian genes appears to be correlated with the GC content of the chromosomal region that contains the genes [12]. This correlation has generally been interpreted as meaning that the codon usage of mammalian genes reflects mutational bias, but a recent report [13] suggests that high GC content increases mRNA levels in mammalian cells. This would mean that selection for gene high expression is the primary factor determining the codon usage bias in this case. Thus, although the correlation between codon usage and nucleotide bias is well documented, the question of whether the nucleotide bias is a cause or a consequence of the biased codon usage remains a matter of debate.

In this study, we examined the patterns of synonymous codon usage that are seen in the genomes of angiosperm plants. It is already known that monocot plant genomes have a higher average GC content than dicot genomes, and that this difference is reflected in an average difference in codon usage between monocots and dicots [14,15]. Here, we focused on the heterogeneity in synonymous codon usage within the rice genome. In particular, we looked for intra-genomic correlations between codon usage and nucleotide bias, and we compared the results found for the rice genes with the results for their homologs in the *Arabidopsis *genome. All of the previous studies of codon usage have focused on either: (i) the comparison of genes within a single genome (typically, a comparison of highly expressed genes and lowly expressed genes); or (ii) differences between genomes, such as differences in codon usage between prokaryotes and eukaryotes, or between thermophiles and mesophiles. Here, we have combined a study of contrasting patterns of codon usage within a genome (rice) with a comparison of homologous gene sequences between two genomes (rice and *Arabidopsis*). This "factorial" design allows for a number of unique controls in the interpretation of the data.

## Results

### Nucleotide content of rice and *Arabidopsis *genes

**Figure 1:**
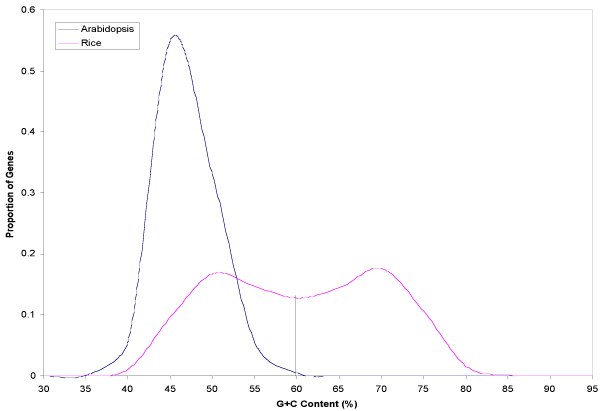
**The distribution of GC contents in rice and *Arabidopsis *genes**. The GC content of the 14,005 rice genes (shown in red) has a bimodal distribution, while the GC distribution of the 25,625 *Arabidopsis *genes (shown in blue) is unimodal. The vertical line (at 60% G+C) shows the point where we separated the rice genes into two classes: high GC and low GC rice genes.

The nucleotide content of rice and *Arabidopsis *coding sequences (expressed as percent GC) is summarized in Figure 1. The Figure shows that there is a distinctly bimodal distribution of GC content among the 14,005 rice genes, which is consistent with previous reports [15,17,23]. In contrast to this, the *Arabidopsis *genes are characterized by a unimodal distribution with a relatively low average value for GC content. In the Figure, the vertical line at 60% GC indicates the point at which we separated the rice genes into two classes: High GC genes and Low GC genes. The average GC content of these two classes, along with the average for all *Arabidopsis *genes is shown in Table 1. From the Table, we can see that the GC content of the *Arabidopsis *genes (44.5%) is comparable to that of the Low GC rice genes (50.1%).

Table 1 also presents the data for the third positions of codons only. In this case, we see the same trends as for all of the codon positions, but the differences are much greater. For instance, the GC content of the third codon positions of the High GC rice genes (80.4%) is almost twice the values for the *Arabidopsis *genes (42.8%). Given that variations in codon usage will affect the third codon position primarily, this result leads us to expect significant differences in codon usage between the two classes of rice genes. We investigated this using Correspondence Analysis (see below).

We also wished to investigate the possible clustering of GC-rich genes within the rice genome. To do this, we took a sample of two rice chromosomes and plotted the GC content at the third codon positions (GC3) against the position of the genes along the chromosome. For comparison, we did the same analysis for the GC3 content of *Arabidopsis* genes along the chromosome. The results (see [33]) show that genes with varying levels of nucleotide composition are interspersed along the chromosome.

### Correspondence analysis

**Figure 2:**
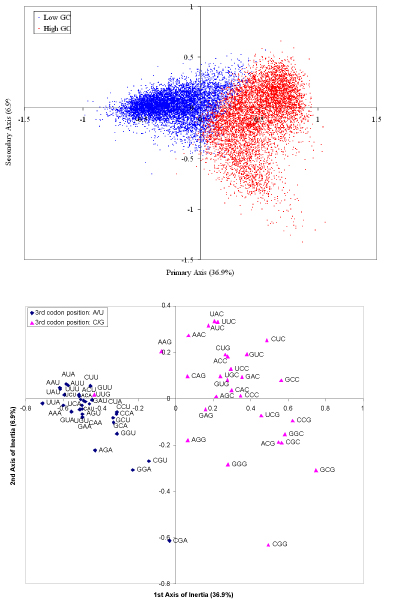
**Correspondence Analysis of relative synonymous codon usage (RSCU) for all 14,005 rice genes**. Panel A. This panel shows the distribution of genes on the primary and secondary axes (accounting for 36.9% and 6.9% of the total variation, respectively). The two classes of genes (High GC and Low GC) are color coded; the high GC genes are shown in red and the low GC genes are shown in blue. Panel B. This panel shows the underlying distribution of codons on the same two axes as shown in Panel A. Codons ending with G or C are shown in red, and codons ending with A or U are shown in blue.

Correspondence analysis [21] was used to explore the variation in Relative Synonymous Codon Usage (RSCU). Since there are a total of 59 synonymous codons (61 sense codons, less the unique methionine and tryptophan codons), this analysis partitions the variation along 59 orthogonal axes, with 41 degrees of freedom. The first axis is the one that captures most of the variation in codon usage, with each subsequent axis explaining a diminishing amount of the variance. In contrast to other types of variance component analysis, such as Principal Component Analysis (PCA), correspondence analysis has the advantage of allowing one to not only show the distribution of genes in the multidimensional space, but also to show the corresponding distribution of synonymous codons (as shown in Figures 2A and 2B). Correspondence Analysis is primarily designed for use with data tables containing counts, e.g., numbers of synonymous codons, whereas PCA is a general method of data reduction that is more suitable for continuous measurement data. Perriere and Thioulouse [22] have provided a critical review of the use of Correspondence Analysis for studies of codon usage.

Figure 2 shows a correspondence analysis of the synonymous codon usage (RSCU) among the rice genes. The origin in Figure 2A represents the average RSCU for all genes, with respect to the first two axes. The distance between genes on this plot is a reflection of their dissimilarity in RSCU, with respect to the two axes. In this case, the two axes account for 36.9% and 6.9% of the variation in the data, respectively. The third axis accounts for approximately 3% of the variation and the remaining axes for even smaller amounts of the variance each. Thus the first axis reflects the primary factor that explains the differences in codon usage among the rice genes. From Figure 2A, we can see that the rice High GC genes (colored red in the Figure) and Low GC genes (colored blue) separate along this primary axis. The corresponding distribution of synonymous codons (see Figure 2B) shows the separation of C/G-ending codons and A/U-ending codons along this same axis. This indicates that the variations in synonymous codon usage among the rice genes are based on the nucleotide content of the genes. The separation of genes on the second axis appears to be largely due to frequency differences in C-ending and G-ending codons among the GC rich genes (see right side of Fig. 2B).

Although the color coding in Figure 2A suggests a general relationship between the nucleotide content of genes and their position on the first axis of the correspondence analysis, it does not give us any statistical measure of this relationship. To do this, we calculated the correlation between the GC content of individual rice genes and their location on the primary axis of the Correspondence Analysis. The results were highly significant (R = 0.96, p < 0.00001), indicating that the variations in codon usage are strongly correlated with the nucleotide content (i.e., GC content) of the genes.

### Effective number of codons

**Figure 3:**
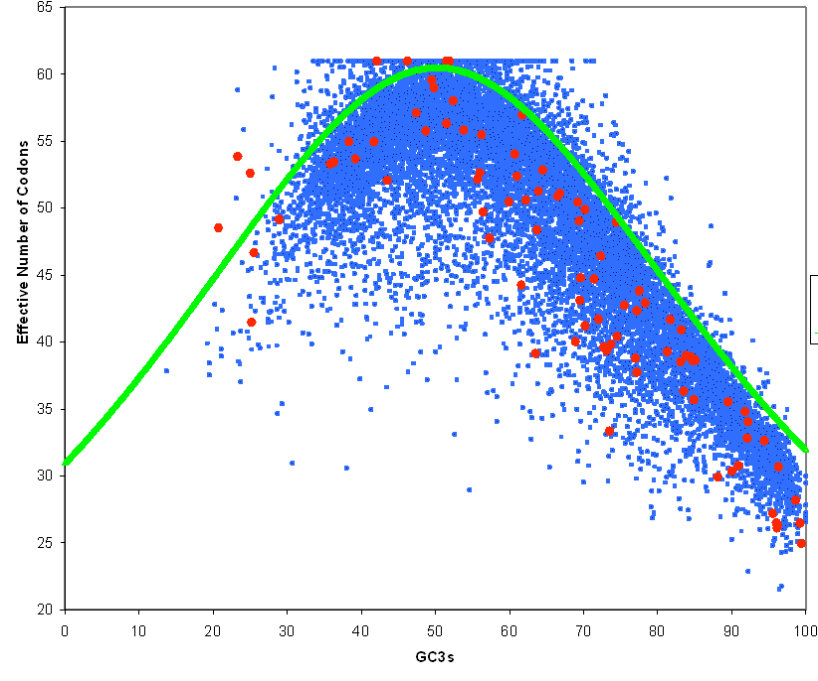
**The effective number of codons (Nc) plotted for 14005 rice genes**. The ribosomal protein genes highlighted in red. The GC(ref) line – shown in green – is the expected position of genes whose codon usage is only determined by the GC content at the third positions of codons (GC3s).

We further investigated the relationship between nucleotide content and codon usage by calculating the effective number of codons for each of the rice genes. The effective number of codons [20] is a measure of the evenness of codon usage among the 61 sense codons. At one extreme is all codons are used equally frequently (given the observed frequencies of amino acids) the effective number of codons is 61. If, at the other extreme, a single codon only is used for each amino acid, then the effective number of codons is reduced to 20. In most cases, the observed number falls somewhere between these extremes. Figure 3 shows the relationship between the effective number of codons (Nc) and the GC content at the third position of each gene (GC3). This Figure also contains a reference line (GCref) showing the expected position of genes whose codon usage is constrained solely by the nucleotide composition at the third codon position [20]. From the Figure, it can be seen that the observed value of Nc tracks the reference line quite closely. This indicates that the nucleotide composition at the third codon position is a major determinant of the effective number of codons. A polynomial line, to the power of 2, that regresses Nc on GC3s (not shown in the figure) fits the data very well (R^2 ^= 0.82, p < 0.00001). Essentially, the effective number of codons decreases as the GC content increases.

### Homologous gene pairs

Although the preceding results clearly establish a strong correlation between nucleotide content and codon usage within the rice genome, they do not tell us which of the two is the causal factor. In an effort to understand the biological basis of these differences in codon usage and nucleotide content within the rice genome, we compared these rice genes with their homologs in *Arabidopsis*. We used a BLAST search to identify 7,160 pairs of homologous genes in rice and *Arabidopsis *(see Methods).

We first calculated the GC content of the homologous gene pairs. Among these gene pairs, we found that the GC content of the *Arabidopsis *homologs remains unimodal, as is shown for the full set *Arabidopsis *genes in Figure 1, whereas the content of the rice homologs remains bimodal, again as shown in Figure 1 for the complete rice data set. Thus, the overall differences between the genomes that are seen in Figure 1 cannot be due to differences in gene content between the two species because they are still present in the homologous gene set.

**Figure 4:**
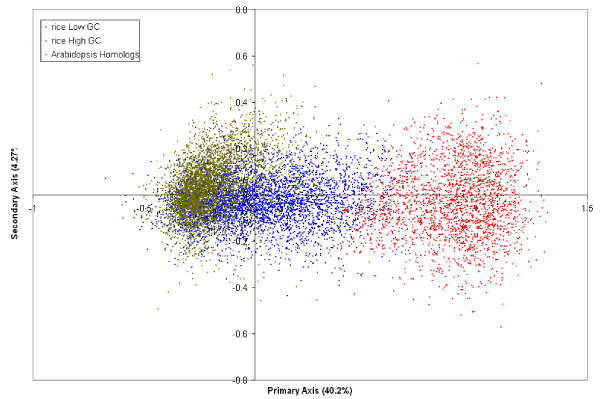
**Correspondence Analysis of relative synonymous codon usage (RSCU) for 7,160 homologous gene pairs from rice and *Arabidopsis***. The Figure shows the distribution of genes on the primary and secondary axes (accounting for 40.2% and 4.2% of the total variation, respectively). High GC rice genes are shown in red; Low GC rice genes are shown in blue; the *Arabidopsis *homologs are shown in yellow.

We then computed relative synonymous codon usage values for the homologous genes and performed a new correspondence analysis. The results are shown in Figure 4. Here we see that the High GC and Low GC rice genes (now defined within the homologous set) again separate along the first axis of the analysis, whereas all of the *Arabidopsis *genes are clustered on the left side of the plot. In other words, the use of homologous genes does not alter the result that we observed in Figure 2. Furthermore, all of the *Arabidopsis *genes have generally similar patterns of codon usage, regardless of whether they are homologs of High GC or Low GC rice genes. This suggests that the divergence in codon usage patterns among rice genes has occurred since the evolutionary divergence of the dicots and monocots approximately 200 million years (My) ago, i.e., over a relatively short evolutionary time.

Although our results suggest that the GC content of the High GC rice genes has increased significantly since the divergence of the monocots and dicots, there remains the formal possibility that, instead, the *Arabidopsis* genes have recently converged toward a common, lower GC content. To distinguish between these possibilities, we extracted 92 homologous sequences from the genome of *Pinus taeda*, and we used these as an out-group to infer the direction of the change. Whereas the High GC rice genes have an average GC content of 66.6 (SE 0.08) and their *Arabidopsis *homologs have an average GC content of 46.0 (SE 0.07), the average GC content of the *P. taeda *homologs is 45.2 (SE 0.04). Thus we can infer that the ancestral condition was similar to that currently seen in *Arabidopsis*.

### Correlation of gene length with GC content

Gene length has previously been shown to be negatively correlated with codon usage in *C. elegans*, *Drosophila *and *Arabidopsis *[25]. We tested to see whether the same relationship holds true for rice genes. We compared the average gene lengths of the two groups of rice genes (High GC and Low GC), as defined in Figure 1. Not only did we find that the High GC genes were shorter, as suggested by previous studies in other species, but the magnitude of this length difference was surprisingly large and highly significant (p < 0.0001). Specifically, the average length of the Low GC coding sequences (1417 +/- 13 bp) is approximately 500 bp larger than the average for the High GC genes (921 +/- 9 bp). Although there is a wide range of individual gene lengths within each class, this highly significant average difference suggests that the length of the rice genes was a significant factor in the evolutionary increase in GC content.

## Discussion

Our survey of codon usage patterns among rice genes shows that there is a wide, multimodal distribution within this genome, in contrast to the much narrower, unimodal distribution of codon usage patterns seen among *Arabidopsis *genes. Our analysis of homologous gene pairs between the two species demonstrates that these contrasting patterns of codon usage cannot be explained by simple differences in gene content between the two genomes. The most parsimonious explanation is that, since the evolutionary divergence of the monocot and dicot plants approximately 200 My ago, there has been a general trend to increase the GC content of the coding sequences within the rice lineage. This increase, however, has occurred in only a subset of the genes. This heterogeneity in nucleotide content is correlated with a large difference in codon usage patterns among the rice genes. A previous study has noted a similar effect of GC content on codon usage in another monocot, *Zea mays *[14], and in the *Gramineae *in general [15].

This demonstration of a strong correlation between the nucleotide composition at the third codon positions (GC3) and codon usage suggests that the variation in codon usage among genes may be due to a mutational bias at the DNA level rather than natural selection acting at the level of mRNA translation. This correlation does not, by itself, prove that the cause is at the DNA level, however. Some inferences about the primary causes can be made by comparing the results seen in rice and *Arabidopsis*. If the large differences in codon usage among rice genes were primarily linked to broad functional classes [26], we would expect to see a parallel pattern among the *Arabidopsis *homologs – but this is not the case when we compare homologous gene pairs between the two species. Specifically, the *Arabidopsis *homologs do not fit into these two classes based on GC content. Moreover, previous studies have provided evidence that codon bias in *Arabidopsis *is correlated with gene expression levels rather than with variations in nucleotide content [27]. These seemingly contradictory results can be reconciled if the patterns of codon usage in both rice and *Arabidopsis *are affected equally by weak translational selection. In the latter case, the absence of strong mutational bias facilitates the detection of the effects of translational selection [27] but, in the rice genome, this translational effect is swamped by the much larger effect of nucleotide bias. This view is consistent with recent findings [7,28] that the relative strength of translational selection can vary widely among genomes.

The question of translational selection versus mutational bias can be approached in a number of other ways. For instance, if codon bias is due to positive selective pressures then we would expect those genes with higher codon bias to have lower rates of synonymous substitution [29]. Such a negative correlation has been observed in bacteria [30], Drosophila [31] and yeast [29]. In contrast to these results, when we compare rice genes with their *Arabidopsis *homologs, we find instead that there is a positive correlation between codon bias and the rate of synonymous substitution. Specifically, there is a higher rate of synonymous substitutions between the High GC rice genes and their *Arabidopsis* homologs than between the Low GC rice genes and their homologs. In order to quantify this relationship between codon bias and divergence rate, we chose a sample of 895 *Arabidopsis* genes from chromosome 4 that had homologs in the rice genome. For each of the 895 rice homologs, we measured the effective number of codons (Nc) and calculated the rate of synonymous substitution (dS). We observed a significant negative correlation (R = -0.27, p < 0.00001). Since the value of Nc is inversely proportional to the level of codon bias, this means that there is a highly significant positive correlation between codon bias and divergence rate in this case. This provides further support for the view that the bias is not due to positive selection for translational efficiency in this case.

Yet another way to distinguish between the effects of mutational bias and translational selection is to compare the nucleotide contents of synonymous and nonsynonymous sites. For instance, if the high GC content at the third codon position of some rice genes were due to translational selection, we would not expect to see any correlation between synonymous and nonsynonymous sites. However, in a previous study [17] we did find correlated patterns of variation in the GC content of non-synonymous sites among rice genes. Finally, the fact that the highly expressed ribosomal protein genes are distributed throughout the entire range of GC contents (see Fig. 3) indicates that the codon bias is not correlated with gene expression level. In summary, it appears that the codon usage of the High GC rice genes is determined primarily by nucleotide bias.

Although we have several lines of evidence that the variations in codon usage are due to the underlying variations in nucleotide content, we still need to explain why some rice genes have become extremely GC-rich while others remain relatively GC-neutral. We found that there is a strong negative correlation between the length of rice genes and their nucleotide content. The reasons why longer genes are more resistant to increases in GC content remain to be elucidated, but one possibility is that the longer genes provide a larger mutational target at the sequence level and that, consequently, they are subject to more purifying selection that counteracts the mutational changes that result in the increased GC content in shorter genes [32]. Another possibility is that the transcription of AT rich genes is, in general, more efficient than that of GC rich genes and this efficiency difference would be more important for longer genes. But if this were the case, we would expect the same forces to be at work among the *Arabidopsis *homologs where we observe the same length difference, but without the associated difference in GC content.

In summary, the simple observation of large differences in codon usage among rice genes might lead us to speculate on functional differences between genes as a basis for the variations in codon usage and GC content. The comparison with homologous sequences from *Arabidopsis*, however, has allowed us to "cross-check" such a prediction and has lead us instead to the conclusion that most of the variation in codon usage among rice genes is not due to positive selection acting on synonymous codon positions. Rather, it is due to a balance between a directional mutational bias, counterbalanced by negative selection acting at all nucleotide positions.

## Methods

### Coding sequence data

14005 rice coding sequences that are longer than 75 codons were obtained from Gramene database [16] and the EMBL as previously described [17]. For the *A. thaliana *coding sequences we used the file containing 25,625 *Arabidopsis *coding sequences (all greater than 75 codons) that we obtained previously [17]. The homologous sequences from *Pinus taeda *(Loblolly pine) were extracted, using BLASTN searches with a cutoff Expect value of 1e-20, from the dataset of 14,198 *P. taeda *sequences in the NCBI Unigene database [34].

### Identification of homologous sequences and computing synonymous substitution rates

Homologous pairs between *O. sativa *and *A. thaliana *were identified by performing BLASTP searches [18] of the rice protein sequences against *Arabidopsis *sequences with a cutoff Expect value of 1e-20. When a rice protein has more than one *Arabidopsis *protein hit, the pair having the lowest Expect value was retained. Using this method, we identified 7,160 homologous gene pairs between the two species, of which 895 gene pairs are rice genes homologous to *Arabidopsis *chromosome 4 genes. In order to see the relationship between codon bias and the evolutionary rate, we used the method of Yang and Nielsen [19] to calculate the synonymous rates for the 895 gene pairs.

### Statistical analyses

Relative synonymous codon usage (RSCU) is the observed frequency of a codon divided by the frequency expected if all synonyms for that amino acid were used equally. An RSCU value close to 1.0 indicates a lack of codon bias. The RSCU was computed for each gene. The RSCU values were then analyzed using correspondence analysis (see below).

The effective number of codons (Nc) is a commonly used measurement to quantify codon usage bias of a gene [20]. The Nc takes a value between 20, when only one synonymous codon is used for each amino acid, and 61, when all codons are uniformly used. Lower Nc values indicate stronger bias. Since Nc is constrained by G+C content of the gene, it is often plotted against GC3s (the frequency of G+C at the third synonymous codon positions) of the gene to investigate patterns of codon usage [20].

Correspondence analysis [21], was used to explore the variation in the 59 RSCU values for each of the 61 sense codons, other than the unique methionine and tryptophan codons. This multivariate statistical method creates a series of orthogonal axes to identify trends that explain the data variation, with each subsequent axis explaining a decreasing amount of the variation. The method, as implemented in CodonW version 1.4 [2], was used in this study. Correspondence analysis assigns ordination for each gene and codon on these axes, and the ordination of the genes and codons can be superimposed. Since the first two axes capture a larger fraction of the variance of the data than any of the other axes, genes and codons were plotted on these two axes only.

## Authors' contributions

H-CW carried out the analyses and drafted the manuscript. DAH conceived of the study, and participated in its design and coordination and helped to draft the manuscript.

## Figures and Tables

**Table 1:** Average GC content of rice and *Arabidopsis *genes.

	All three codon positions	Third codon positions only
		
High GC rice genes (n = 6291)	67.4 ± 0.05	80.4 ± 0.14
Low GC rice genes (n = 7714)	50.1 ± 0.06	52.7 ± 0.12
*Arabidopsis *genes (n = 25625)	44.5 ± 0.02	42.8 ± 0.04

The values shown are percentages of G+C. Standard errors are included.

High GC rice genes are defined as those that have a G+C content equal or greater than 60%. Low GC rice genes have a G+C content less than 60%.
